# A Remote Sensing Approach for Displaying the Changes in the Vegetation Cover at Az Zakhnuniyah Island at Arabian Gulf, Saudi Arabia

**DOI:** 10.1155/2022/2907921

**Published:** 2022-03-17

**Authors:** Wafa'a A. Al-Taisan

**Affiliations:** Department of Biology, College of Science, Imam Abdulrahman Bin Faisal University, P.O. Box 1982, 31441 Dammam, Saudi Arabia

## Abstract

In the terrestrial ecosystem, vegetation is the important component of exchanging of water and energy in biogeochemical and climate cycle. A study was conducted to detect the vegetation cover change at Az Zakhnuniyah island by using remote sensing techniques. It includes vegetation analysis using normalized difference vegetation index (NDVI) while comparing with climatological data including temperature, humidity, and precipitation. A clear trend was seen in climatological parameters where temperature and humidity were rising decade by decade although NDVI did not show. In addition, increasing soil salinization over the years was observed when soil salinity index was used. NDVI-based long-term decadal analysis on vegetation cover based on Landsat surface reflectance data showed increase of vegetation cover which was also linked to precipitation trends. Also, the short-term demi-decadal comparison using PROBA-V showed the vegetation cover reduction between 2015 and 2019. Nevertheless, the sea level surrounding the island also showed an increasing trend of 0.34 cm/y, which could be the cause of inundation in some parts of the island in future. Furthermore, all these trends need to be observed in entirety as many of those trends can be interlinked.

## 1. Introduction

In smaller marine islands, it is necessary to understand impacts of natural and anthropogenic factors by monitoring vegetation changes. According to the classic island biogeography theory, small islands support very few species either due to the absence of suitable habitats for long-term survival or due to intense or frequent disturbances [1]. Climate change induces large-scale changing of plant growth patterns and morality. Increased warming trends predictions show that it will cause shifting in plant distribution. Kelly and Goulden [2] found that global climate change in recent time affected the distribution of plant. Anthropogenic activities affected vegetation cover and caused spatiotemporal variations with the combined effect of climate change [3].

Remote sensing (RS) techniques used in this study include utilising Landsat products like surface reflectance products for NDVI, salinity indices, and land surface temperature (LST). This mostly employs band math, algorithm for LST, and threshold technique for obtaining land cover from NDVI. Whereas, other model-based and long-term climate datasets are used for other climatological parameters in this study. The characteristics of NDVI significantly reduce the variations produced in the remote sensing process in terms of radiometric, spectral properties, and atmospheric conditions. However, some surface features such as snowing and nonvegetated surfaces are not exhibited properly by NDVI [4]. The use of NDVI in understanding temporal change in various ecosystems is important, as annual averages, maximum, and minimum NDVI values can provide unified understanding of photosynthetic activity [5]. Hence, identifying trend using NDVI temporally not only is effective for determining but also quantifying change in ecosystem attributes locally as well as globally [6]. Overall, vegetation indices like NDVI serve as a robust representative of vegetation activity [4].

Furthermore, NDVI studies show that low NDVI between 0.01 and 0.3 index values indicate weak or stressed vegetation especially in regions with arid climatic conditions [7], which is expected to be in our study area too. In addition, NDVI threshold methods include different threshold values based on which various surface features can be effectively extracted and vegetation densities can be identified, which is a part of vegetation analysis [8]. Salinity index (SI) has shown best results among other soil salinity indices (0.83*R*^2^) when comparison was done with satellite images derived indices values with field electrical conductivity samples [9]. Thus, it was chosen for use in this study.

For tidal constituent amplitude data of the study area, the HAMTIDE (Hamburg direct data Assimilation Methods for TIDEs) model was used. It aims at direct reduction of model deficiencies and inaccuracies of recordings by using an iterative method for solving equations, and simultaneously, model correction based on inferring physics from the datasets is performed. The use of dynamic residuals helps in detection of errors in the model, e.g., bathymetry, parameterization of dissipation [10]. The corals were mapped using the UNEP-WCMC dataset of global distribution of coral reefs. Also, seagrasses datasets were obtained from Global Distribution of Seagrass Biome, a UNEP-WCMC dataset [11]. Changes in vegetation reflectance is a useful basis for understanding feedbacks to global warming as the reflectance of vegetation in red and near-infrared (NIR) spectrum depends on the chemical and physical components in plants which provide a representation of photosynthetic activity [12].

The coastal salt marshes are usually dominated by halophytes that bioaccumulate, and these halophytic communities can help in phytoremediation for mitigating of soil salinity [13]. Moreover, salt marshes provide environmental benefits as they are highly productive ecosystems, and the halophytic flora of salt marsh can help in reclamation of salt-affected land and along with that provides economic benefits as they possess fodder, timber, and medicinal advantages [14]. The goal of the present study was to track the vegetation changes, depth of marsh surface, tidal changes, climatological parameters, and soil salinity estimation by remote sensing. The study also performed the mapping of plant communities for understanding the temporal changes.

## 2. Materials and Methods

**Figure 1:**
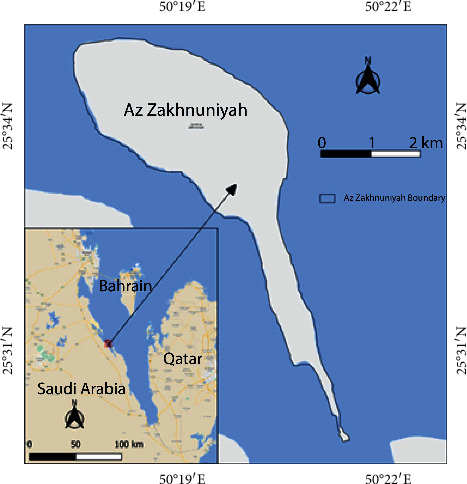
Study area map showing Az Zakhnuniyah island. Base map credits: google maps.

Az Zakhnuniyah is an island in Arabian Gulf governed under Saudi Arabia which is located in west and about 380 km east from Riyadh (Figure 1). The total area of the island is 13.35 sq. km (1335 ha). It is mostly Barren; however, many parts are covered with halophytic/xerophytic plants. The area is characterized by hot desert (arid) climate (BWh) under the Köppen–Geiger climate classification system both in the current period and future, based on models [15]. On the west coast of the island, there are salt marshes present containing water, mostly seasonal or tidal; however, some parts of the marsh have permanent water, mostly on edges as observed from PROBA-V fractional water cover; whereas, earlier, it had water in land as observed in 2015 water cover.

The methodology includes a geospatial and remote sensing analysis of the island in terms of long-term environmental, climatological, and vegetational aspects. It uses satellite datasets of different satellites at defined intervals. Climatological parameters such as LST (land surface temperature), precipitation, and humidity are usually taken as averages over an annual period, i.e., 1999-2000 (2000), 2010-2011 (2011), and 2019-2020 (2020). Thus, year proceeding the reference year is taken in all analyses, where these 3 years are used in order to observe the effects of climatological factor in first month of each year. The methodology is given in Table 1.

### 2.1. Calculating NDVI and General Land Cover

NDVI shows the vegetation presence of surface based on utilising difference in reflectance of surface features as shown in the following equation.

(1) $$\begin{array}{c} \text{NDVI}=\frac{\left(\text{NIR}-\text{red}\right)\;}{\left(\text{NIR}+\text{red}\right)}. \end{array}$$

NDVI utilises reflectance in near infrared (NIR) and red band based on which vegetation analysis can be performed. Positive values up to 1 indicate some presence of vegetation, whereas 0 and further negative values indicate absence of vegetation [16]. Landsat surface reflectance (level 2) products were used to obtain NDVI averages for all years. Using the NDVI threshold method, thresholds for different land covers were estimated using mean NDVI values found from a study in similar climate in Saudi Arabia [17] based on which general land cover maps were prepared.

### 2.2. Biomass Density Estimation

Due to lack of field information of biomass density of plant communities, remote sensing based analysis was performed. The IPCC Tier-1 Global Biomass Carbon Map dataset (2000) was used for estimating biomass carbon density for the island. The spatial resolution of the dataset is 1 km, and therefore, for better visualization, IDW (inverse distance weighting) interpolation was carried out by point sampling of the grid cells falling in the island area, and a smooth raster was obtained.

### 2.3. Depth of Marsh Surface

ALOS-PALSAR (Advanced Land Observation Satellite-Phased Array type L-band Synthetic Aperture Radar) 12.5 m spatial resolution-DEM data were used to measure depth of the marsh surface. Synthetic Aperture Radar (SAR) data from ALOS-PALSAR has been used earlier for its application in obtaining water depths in fresh-water marsh, which can help understand spatial distribution of habitats harboring various communities [18]. The raw data generally had sinks which were filled (processed) for more accuracy in estimation. Here, the DEM was subsetted (after fill sink was performed) for the island. However, this was verified with GEBCO (General Bathymetric Chart of the Oceans) gridded bathymetry data.

### 2.4. Tidal Range

Tidal range is simply the height difference between high tide and low tide. However, it varies daily; therefore, we have used the mean tidal range, i.e., difference between average high tide and average low tide. The M_2_ lunar constituent is the major constituent of semidiurnal tides. Apart from that, principal solar constituent S_2_ and larger lunar elliptic constituent N_2_ are responsible for tides. Another constituent includes lunar elliptical second-order constituent 2N_2_. In this analysis, the HAMTIDE model was used to obtain the tidal constituents for estimation of tidal range which has spatial resolution of 7.5′ (minutes). These constituents' values are in amplitude (cm) which are summed up to obtain the total amplitude for further obtaining tidal range [19].

Also, to understand any long-term sea level changes, the altimetry-based sea level trend is shown in figure 9. The altimetry data are based on SSHA (sea surface height anomalies) data of TOPEX/POSEIDON, Jason 1, 2, and 3, and other altimetry-based satellites provided by University of Colorado Boulder.

### 2.5. Percentage of Soil Salinity Estimation by Remote Sensing

The percentage of soil salinity was estimated by the general salinity index (SI). For soil salinity analysis, Landsat surface reflectance data were used for 2000 and 2020 (January months) and ASTER surface reflectance data were used instead of Landsat (due to availability issues) for 2011 (January month). Hence, SI obtained using ASTER surface reflectance values was scaled for Landsat for better comparison.

SI tilizes the green and red bands [20]. The computation equation for SI is

(2) $$\begin{array}{c} \text{SI}=\sqrt{\text{green}\times \text{red}}. \end{array}$$

### 2.6. Mapping Plant Communities for Understanding Temporal Change

Most of the area of the island is covered by halophytic and xerophytic plants which were also detected by obtaining fractional shrubland (mainly halophytic and xerophytic plants) cover from PROBA-V based Copernicus land cover service, and it shows the cover of 2015 and 2019 for comparison. In addition, the general land cover maps derived from NDVI annual averages also provide the land cover statistics covered not only by mainly shrubland, i.e., halophytic and xerophytic plants but also medium to dense vegetation (although very less area).

### 2.7. Climatological Parameters: Temperature, Precipitation, and Humidity

In addition, specific humidity maps were instead interpolated from CFS reanalysis data due to lower resolution of CFS data. Daytime land surface temperature (LST) was derived from Landsat TOA (Top of the atmosphere) reflectance products. The monowindow algorithm provides the capability to obtain LSTs by using emissivity, transmittance, and effective atmospheric temperature from Landsat thermal bands and metadata [21]. The LSTs were annual averages of respective years mentioned earlier. Precipitation was obtained from CHIRPS (Climate Hazards Group InfraRed Precipitation with Station data) with 0.05 spatial resolution. As this spatial resolution was low, the precipitation maps were also interpolated using IDW interpolation just as in the case of specific humidity.

## 3. Results and Discussion

### 3.1. Vegetation Analysis

**Figure 2:**
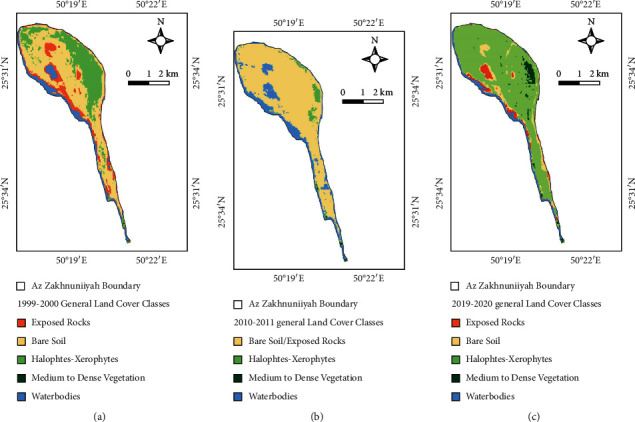
General land cover maps (a) based on 1999-2000 NDVI, (b) based on 2010-2011 NDVI, and (c) based on 2019-2020 NDVI.

General land cover maps prepared using the NDVI threshold technique is shown Figure 2. Vegetation cover is the highest (>70%) during 2019-2020 with halophytic and xerophytic plants as well as medium to dense vegetation in some parts of the island. 2010-2011 had the lowest vegetation cover in general (<5%), whereas 1999-2000 had moderate vegetation cover compared to other years (Table 2). Nonvegetation includes surface features such as waterbodies, bare soil, and exposed rocks which was the highest in 2010-11. Waterbodies cover the least area in 2019-2020 compared to other years (Figure 2).

In this study, 3 years were taken as reference years for vegetation analysis 2000, 2011, and 2020, of which annual NDVI averages are used for comparison. The mean, max., and min. values of NDVI are given in Table 3.

**Figure 3:**
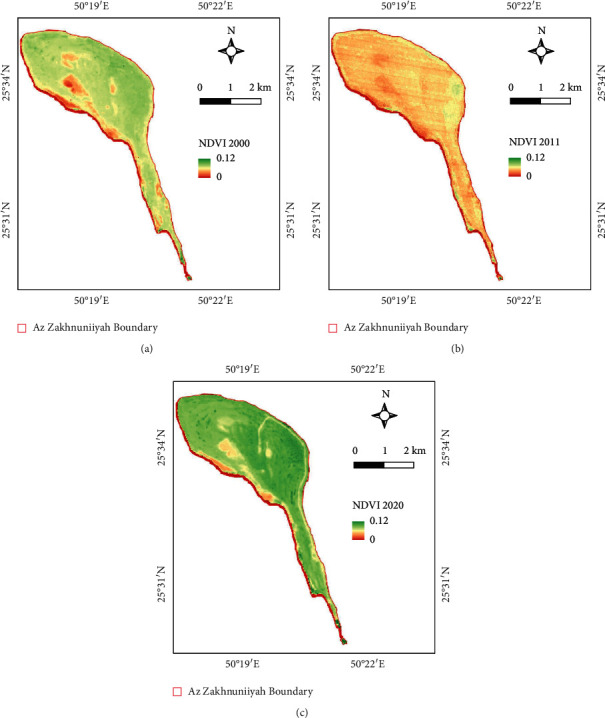
NDVI map for years (a) 2000, (b) 2011, and (c) 2020.

The NDVI results show that the average NDVI of 2000 was higher than 2011, but again in 2020, NDVI increased, respectively (Figures 3(a)–3(c)). However, the mean and max. average (mean of the year) values indicate the presence of only sparse vegetation like halophytic and xerophytic plants (Table 3). In addition, the negative values are mostly seen in the fringes of the island where permanent or seasonal water is present. In 2000, the salt marsh shows significant negative values in the north which indicates water presence which is not present that much significant in later years (Figures 3(a)–3(c)).

### 3.2. Biomass Density Estimation

**Figure 4:**
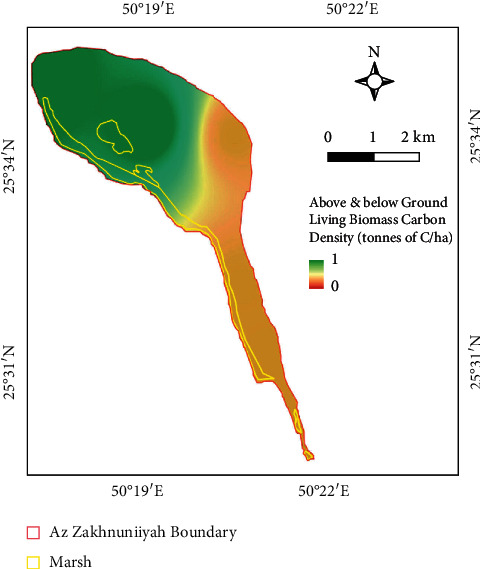
Above and below ground living biomass carbon density map. Units in tonnes of carbon/hectare.

The northern part, where salt marsh and area near the coast, had higher biomass accumulation (up to 1 tonne of carbon/hectare) than the remaining island where negligible values were observed (Figure 4). The mean biomass density of the entire island was 0.52 tonnes of C/ha (Table 4). Although, this is much less compared to many parts of the world where it can way go above 200 tonnes of C/ha, especially in tropical areas [22]. Moreover, global forest biomass has total carbon density around 81.7 tonnes of C/ha based on a study which had utilized a comprehensive data of around 8800 biomass plots globally [23].

### 3.3. Depth of Marsh Surface

**Figure 5:**
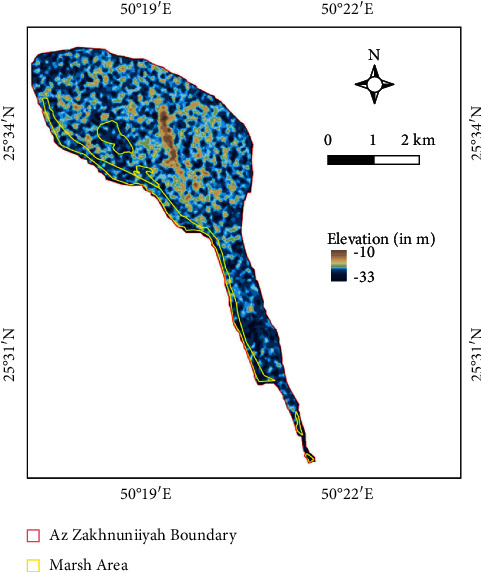
Digital elevation model map showing elevation in m. Note: the fill sink operation was performed on this DEM later to remove many negative values which cause accuracy problems. The raw DEM is shown for better visualization.

**Figure 6:**
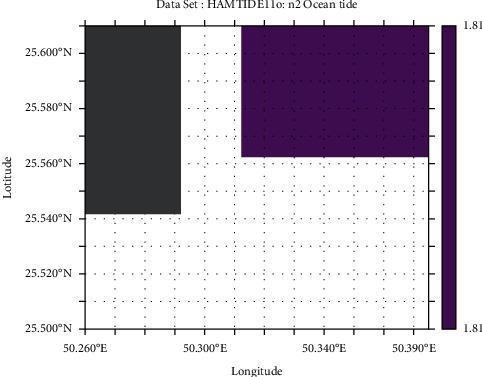
Amplitude in cm of N_2_ tidal constituent of study area.

**Figure 7:**
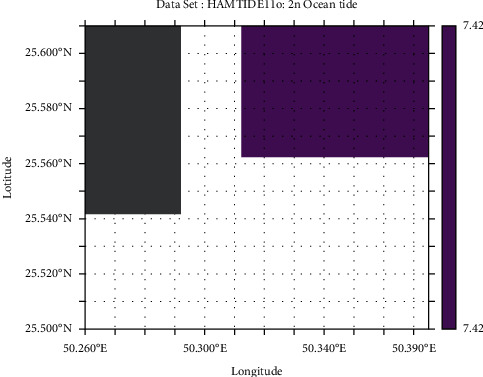
Amplitude in cm of 2N_2_ tidal constituent of study area.

**Figure 8:**
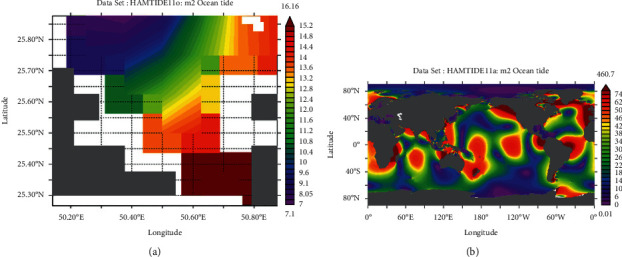
Amplitude in cm of M_2_ tidal constituent of (a) larger local area surrounding the island for comparison and (b) global coverage for comparison.

**Figure 9:**
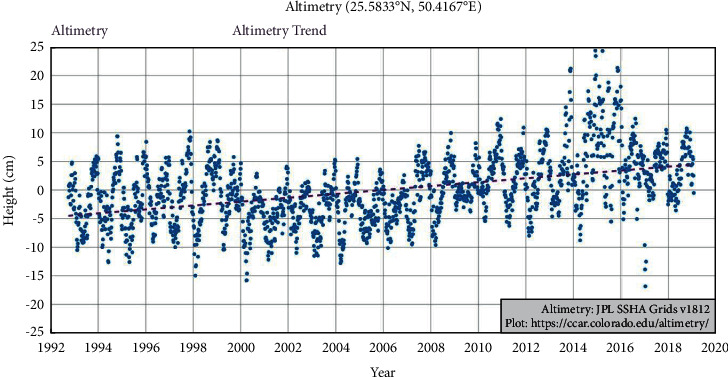
Satellite altimetry data plotted for year-wise showing sea level changes (in cm).

From the GEBCO data, the mean depth of marsh surface was 0.7 m (−0.7 m elevation), which was still in range with average marsh surfaces around the world in general; however, the bathymetry data resolution was very low compared to DEM (ALOS-PALSAR). Hence, the mean depth of marsh surface was estimated to be 1.43 m (−1.43 m elevation) based on DEM (Figure 5). The amplitude (in cm) of N_2_ and 2N_2_ of different tidal constituents is shown in detail in HAMTIDE maps (Figures 6 and 7) for the immediate area surrounding the island; whereas, for comparison about the tidal range variation, it is shown in amplitude (in cm) of M_2_ tidal locally (Figure 8(a)) and globally (Figure 8(b)). However, as amplitude is for only one tide, therefore, the tidal range is twice the amplitude. Therefore, the average tidal range of the island area is estimated to be 45.42 cm (Table 5). It shows a general trend of rise in sea level by 0.34 cm/y from 1992 to 2020 (Figure 9). It can cause future inundation on low-lying parts of the island keeping in mind elevation, tidal amplitude, and sea level rise as a study indicated 2 m rise in sea level can submerge many islands in the eastern coastal zone of Saudi Arabia in the Arabian Gulf [24].

**Figure 10:**
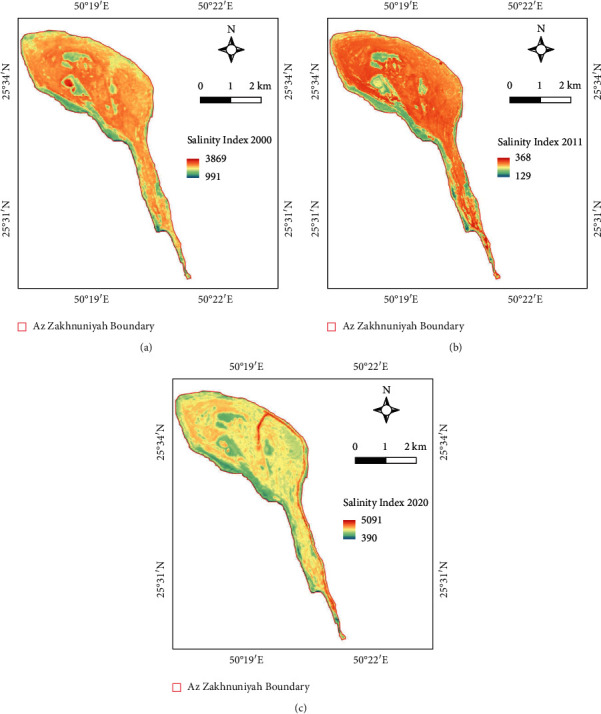
Salinity index (SI) map of (a) 2000, (b) 2011, and (c) 2020.

The general trend shows significantly increasing percentage of soil salinity on the island between 2000 and 2020, in a period of two decades. However, SI showed a higher average value in 2011 than that of salinity in 2020, though the maximum salinity was observed in 2020 (Table 6). Thus, increasing soil salinization is the problem identified using these results and may increase in future. The percentage of soil salinity was found to be low in the halophytes of salt marsh area in all years (Figures 10(a)–10(c)).

### 3.4. Mapping Plant Communities for Understanding Temporal Change

**Figure 11:**
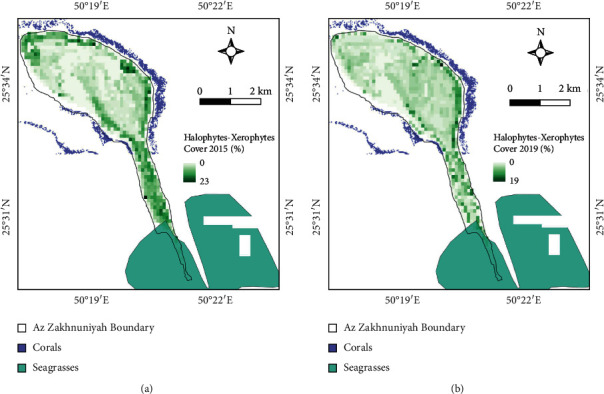
Plant communities map including corals, seagrasses, and halophytic/xerophytic plants. Halophytic/xerophytic plants cover fraction for years (a) 2015 and (b) 2019.

The vegetation communities on the island were found to be halophytic/xerophytic plants mainly along with mixed seagrass species mostly in southern part of the island (Figure 11). The available seagrasses are *Halophila ovalis*, *Halophila stipulacea*, and *Halodule uninervis* based on GBIF (Global Biodiversity Information Facility). In the Arabian Gulf region, almost 77 species of corals of various genus such as *Acanthastrea*, *Acropora*, *Favia*, *Goniopora*, *Montipora*, *Pavona*, *Porites*, and *Turbinaria* are found based on Corals of the World portal data. These datasets were compared with the extent provided by UNEP. The mean fractional cover of halophytic/xerophytic plants decreased between 2015 and 2019 (Table 7).

### 3.5. Climatological Parameters: Temperature, Precipitation, and Humidity

**Figure 12:**
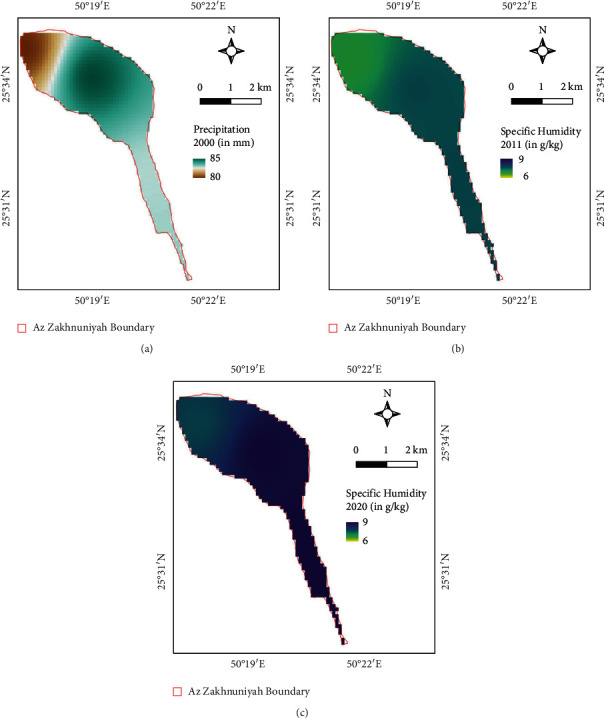
Specific humidity map (in g/kg) of study area for (a) 2000, (b) 2011, and (c) 2020.

Specific humidity also showed a clear increasing trend from 2000 to 2020 (Table 8; Figures 12(a)–12(c)). This means that increasing water vapor is also linked with increasing LSTs, and these indicators show similar trend with projections of global temperature and specific humidity increase under climate change in 21^st^ century [25]. There is a clear trend for LSTs as the temperature seems to be increased over the years. However, precipitation in a semiarid climate as usual is very low, which is also seen here (<100 mm/y) for all years. The lowest precipitation was observed in 2011. But over the years, there is no major difference between 2000 and 2020 (Table 8). This is also in accordance with the mean NDVI on the island that NDVI was the lowest in 2010-2011 year where precipitation was also the lowest among the study years (Tables 3 and 8) because NDVI dynamics are highly associated with water availability based on the behavior of climatological parameters such as temperature and precipitation [26].

**Figure 13:**
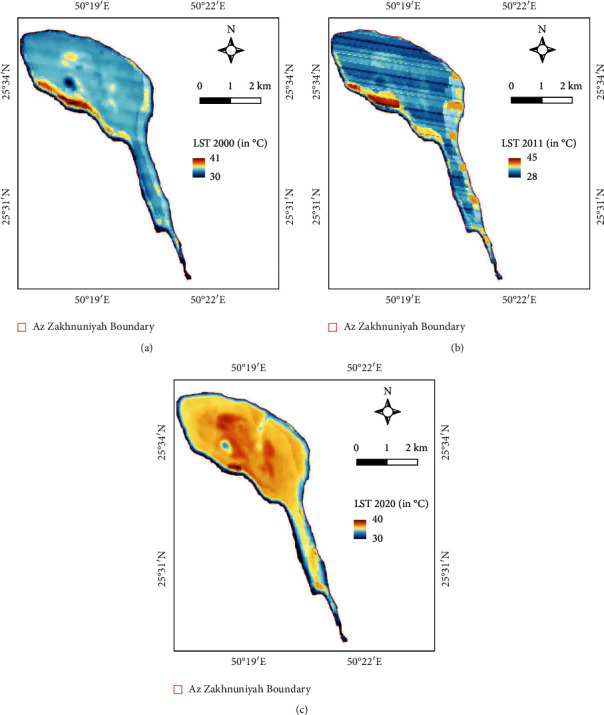
LST map (°C) of study area for (a) 2000, (b) 2011, and (c) 2020.

**Figure 14:**
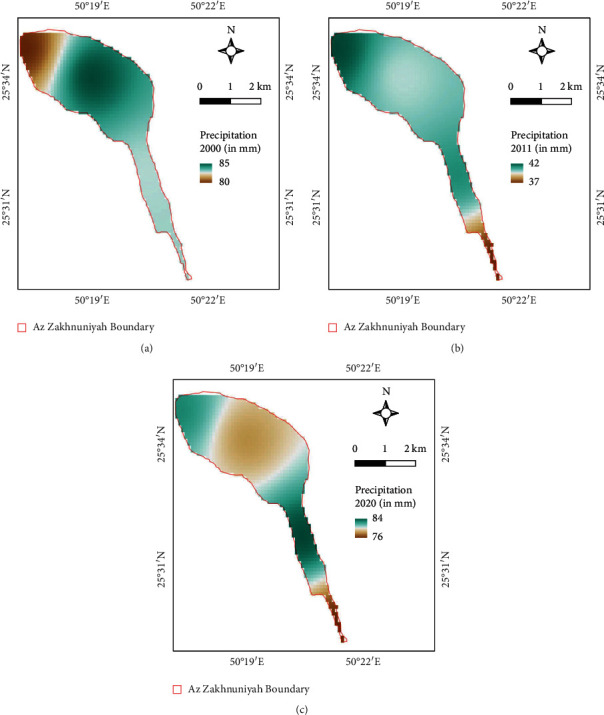
Precipitation map (in mm) of study area for (a) 2000, (b) 2011, and (c) 2020.

LSTs were slightly higher in the northern part salt marsh area in 2000 (Figure 13(a)) as well as in 2011, but the line-like patches are due to scan line errors in Landsat-7 instruments there for it is evident in the mean LST map (Figure 13(b)) as well, but it is understandable due to data availability. Whereas, in 2020, the northern central part of the island has higher LSTs compared to other areas (Figure 13(c)). Based on precipitation maps, in 2000, the precipitation was higher in the northern central part than that of the precipitation of the top northwest part of the island (Figure 14(a)). Whereas, in 2011, the top of northwest part had higher precipitation compared to the south (Figure 14(b)). In 2020, the north central and northern part had lower precipitation compared to the central strip of the island (Figure 14(c)).

## 4. Conclusion

Our results based on annual averages of vegetation and salinity indices along with climatological parameters show a comprehensive assessment of vegetation on the island. Clear trends include increasing the percentage of soil salinity, LSTs, and humidity between 2000 and 2020 in a period of two decades which resonates global trends relating to climate change. However, in a semiarid region where there is low rainfall, there was no clear trend in terms of precipitation; however, a link between precipitation and vegetation cover is observed where years with lower precipitation show lower NDVI generally. It is also seen that halophytic/xerophytic plants cover and other sparse to dense vegetation area increased although gradually in two decades. However, more trends spanning over 40–50 years can provide a more detailed analysis in semiarid regions. It is recommended to employ the use of hyperspectral imagery for mapping individual species of interest such as halophytic species while keeping in mind the spatiotemporal availability of hyperspectral datasets.

## Figures and Tables

**Table 1:** Summary of methodology.

	Objective/subobjectives	Analysis type	Method	Raw data/data source
1	Vegetation analysis	Decadal	NDVI	Landsat surface reflectance
2	Biomass density	Single annual	Biomass carbon density	IPCC-CDIAC
3	Depth of marsh surface	Long term	DEM-bathymetry combination based	ALOS-PALSAR and GEBCO
4	Tidal range	Long term	M_2_ lunar constituent amplitude	ICDC-HAMTIDE
5	Soil salinity	Decadal	Soil salinity index	Landsat/Aster surface reflectance
6	Plant communities	Demi-decadal	Fractional cover; coral and seagrass datasets	PROBA-V Copernicus; UNEP; GBIF
7	Temperature	Decadal	Land surface temperature	Landsat TOA reflectance
8	Humidity	Decadal	Mean specific humidity	CFS reanalysis
9	Precipitation	Decadal	Quasiglobal rainfall dataset	CHIRPS

**Table 2:** NDVI-based land cover/vegetation cover area (ha) and percentage for comparison years.

Area (in hectares, ha)	1999-2000	2010-2011	2019-2020
Halophytic and xerophytic plants cover	325.6 (24.3%)	44.5 (3.33%)	900.2 (67.38%)
Medium to dense vegetation	0.5 (0.04%)	2.5 (0.19%)	40.5 (3.03%)
Total vegetation	326.1	47.0	940.6
Nonvegetation	1009.9	1289.0	395.3

**Table 3:** Year-wise NDVI values statistics comparison.

NDVI	2000	2011	2020
Mean	0.015	0.010	0.018
Max.	0.12	0.11	0.14
Min.	−0.38	−0.49	−0.46
SD	0.035	0.027	0.048

**Table 4:** Basic statistics of above and below ground living biomass carbon density of the island.

Biomass carbon density (tonnes of C/ha)	
Mean	0.52
Max.	1.0
Min.	0.0
SD	0.36

**Table 5:** Tidal constituents and their respective tidal amplitudes (cm) surrounding the island.

Tidal constituent	Tidal amplitude (cm)
M_2_	11.12
S_2_	2.36
N_2_	1.81
N_2_	7.42
Total	22.71
Tidal range	45.42

**Table 6:** Statistics comparison table for SI of the island for 2000, 2011, and 2020.

SI	2000	2011	2020
Mean	2440	2828	2658
Max.	3468	3670	5090
Min.	991	1291	393
SD	322	400	533

**Table 7:** Statistics comparison fractional cover (%) of halophytic/xerophytic plants between 2015 and 2019.

Fractional cover	2015 (%)	2019 (%)
Mean	4.4	2.97
Max.	23	19
SD	4.6	2.9

**Table 8:** Statistics comparison table for climatological parameters for 2000, 2011, and 2020.

Mean	2000	2011	2020
LST (°C)	34.14	34.20	34.79
Sp. humidity (g/kg)	6.76	7.4	8.38
Precipitation (mm)	82.56	39.89	81.01

## Data Availability

The data used to support the findings of this study are included within the article.
